# Effects of a Novel Approach to Reducing Costly Hospitalizations of Nursing Home Residents

**DOI:** 10.1093/geroni/igaa057.2741

**Published:** 2020-12-16

**Authors:** Ruth Tappen, David Wolf, Karen Southard

## Abstract

The reduction of preventable hospitalizations from long term care facilities has been identified by CMS as an important measure of quality, both in terms of resident outcomes and nursing home performance. As many as one-quarter of individuals admitted to nursing homes from acute care are rehospitalized within the month placing them at high risk for increased falls, delirium, skin breakdown, nosocomial infection and the like and costing an estimate $4.3 billion annually. To address this well documented threat to care quality, CMS has imposed significant penalties for excessive readmissions on facilities with high rehospitalization rates. An important contributor to these preventable readmissions is resident and family insistence on the transfer. Early efforts to reduce potentially preventable hospitalization of nursing home residents focused on developing systems to identify and respond to acute changes in condition before hospitalization becomes necessary. Reports from facility staff, however, brought to our attention the additional problem of resident and family insistence on transfer despite provider recommendations to the contrary. A series of funded studies to understand this problem, develop a solution and test the effectiveness of this solution will be reported by an interdisciplinary team. We begin with description of the development and clinical trial of Go to the Hospital or Stay Here?, an evidence-based, patient-centered decision aid, with individual residents and families, followed by a pilot test of facility-wide implementation; then an eight state regional dissemination supported by CMS and participating states and finally a discussion of best practices for effective implementation of the Guide.

